# Regularization of Ill-Posed Point Neuron Models

**DOI:** 10.1186/s13408-017-0049-1

**Published:** 2017-07-14

**Authors:** Bjørn Fredrik Nielsen

**Affiliations:** Faculty of Science and Technology, Norwegian University of Life Sciences, P.O. Box 5003, Ås, 1432 Norway

**Keywords:** Point neuron models, Ill-posed, Regularization, Existence

## Abstract

Point neuron models with a Heaviside firing rate function can be ill-posed. That is, the *initial-condition*-to-*solution* map might become discontinuous in finite time. If a Lipschitz continuous but steep firing rate function is employed, then standard ODE theory implies that such models are well-posed and can thus, approximately, be solved with finite precision arithmetic. We investigate whether the solution of this well-posed model converges to a solution of the ill-posed limit problem as the steepness parameter of the firing rate function tends to infinity. Our argument employs the Arzelà–Ascoli theorem and also yields the existence of a solution of the limit problem. However, we only obtain convergence of a subsequence of the regularized solutions. This is consistent with the fact that models with a Heaviside firing rate function can have several solutions, as we show. Our analysis assumes that the vector-valued limit function **v**, provided by the Arzelà–Ascoli theorem, is threshold simple: That is, the set containing the times when one or more of the component functions of **v** equal the threshold value for firing, has zero Lebesgue measure. If this assumption does not hold, we argue that the regularized solutions may not converge to a solution of the limit problem with a Heaviside firing function.

## Introduction

In this paper we analyze some mathematical properties of the following classical point neuron model:

1 $$ \begin{aligned} &\tau_{i} u'_{i}(t)= -u_{i}(t) + \sum_{j=1}^{N} \omega_{i,j} S_{\beta} \bigl[u_{j}(t)-u_{\theta} \bigr] + q_{i}(t), \quad t \in(0,T], \\ &u_{i}(0)=u_{\mathrm{init},i}, \end{aligned} $$

for $i=1,2,\ldots,N$, where

$$\begin{aligned} &u_{i}(t) \in \mathbb {R}, \quad t \in[0,T], i=1,2,\ldots,N, \\ &q_{i}(t) \in \mathbb {R}, \quad t \in(0,T], i=1,2,\ldots,N, \\ &u_{\mathrm{init},i} \in \mathbb {R}, \quad i=1,2,\ldots,N, \\ &u_{\theta} \in \mathbb {R}, \\ &\omega_{i,j} \in \mathbb {R}, \quad i,j=1,2,\ldots,N, \\ &\tau_{i} \in \mathbb {R}_{+}, \quad i=1,2,\ldots,N, \\ &\beta=1,2,\ldots,\infty, \\ &S_{\beta}[x] \mbox{ is an approximation of the Heaviside function } H[x], \\ &S_{\infty}[x]=H[x]. \end{aligned}$$

Here, $u_{i}(t)$ represents the unknown electrical potential of the *i*th unit in a network of *N* units. The nonlinear function $S_{\beta}$ is called the *firing rate function*, *β* is the *steepness parameter* of $S_{\beta}$, $u_{\theta}$ is the *threshold value for firing*, $\{ \omega_{ij} \}$ are the connectivities, $\{ \tau_{i} \}$ are membrane time constants and $\{ q_{i}(t) \}$ model the external drive/external sources, see, e.g., [1–3] for further details. The system of ODEs (1) is also referred to as a voltage-based model or Hopfield model (due to Hopfield [4]).

By employing electrophysiological properties one can argue that it is appropriate to use a steep sigmoid firing rate function $S_{\beta}$. But due to mathematical convenience the Heaviside function is also often employed, see, e.g., [5–8]. Unfortunately, when $\beta= \infty$ the *initial-condition*-to-*solution* map for (1) can become discontinuous in finite time [9]. Such models are thus virtually impossible to solve with finite precision arithmetic [10, 11]. Also, in the steep but Lipschitz continuous firing rate regime, the error amplification can be extreme, even though a minor perturbation of the initial condition does not change which neurons that fire. It is important to note that this ill-posed nature of the model is a fundamentally different mathematical property from the possible existence of unstable equilibria, which typically also occur if a firing rate function with moderate steepness is used. See [9] for further details.

The solution of (1) depends on the steepness parameter *β*. That is,

$$ u_{i}(t) = u_{\beta,i}(t), \quad i=1,2,\ldots,N, $$

and the purpose of this paper is to analyze the limit process $\beta\rightarrow\infty$. This investigation is motivated by the fact that the stable numerical solution of an ill-posed problem is very difficult, if not to say impossible, see, e.g., [10, 11]. Consequently, such models must be regularized to obtain a sequence of well-posed equations which, at least in principle, can be approximately solved by a computer. Also, steep firing rate functions, or even the Heaviside function, are often used in simulations. It is thus important to explore the limit process $\beta\rightarrow\infty$ rigorously.

In Sects. 3 and 4 we use the Arzelà–Ascoli theorem [12–14] to analyze the properties of the sequence $\{ \mathbf{u}_{\beta} \}$, where

2 $$ \mathbf{u}_{\beta} (t)= \bigl( u_{\beta,1}(t), u_{\beta,2}(t), \ldots, u_{\beta,N}(t) \bigr) ^{T}. $$

More specifically, we prove that this sequence has at least one subsequence which converges uniformly to a limit

$$ \mathbf{v}(t)= \bigl(v_{1}(t), v_{2}(t), \ldots, v_{N}(t) \bigr)^{T}, $$

and that this limit satisfies the integral/Volterra version of (1) with $S_{\beta}=S_{\infty}$, provided that the following set has zero Lebesgue measure:

$$ \bigl\{ s \in[0,T] \mid\exists i \in\{1,2,\ldots, N \} \mbox{ such that } v_{i}(s) = u_{\theta} \bigr\} . $$

It is thus sufficient that this set is finite or countable; see, e.g., [13]. Furthermore, in Sect. 7 we argue that, if **v** does not satisfy this threshold property, then this function will not necessarily solve the limit problem.

According to the Picard–Lindelöf theorem [15–17], (1) has a unique solution, provided that $\beta< \infty$, and that the assumptions presented in the next section hold. In Sect. 5 we show that this uniqueness feature is not necessarily inherited by the limit problem obtained by employing a Heaviside firing rate function. It actually turns out that a different subsequence of $\{ \mathbf{u}_{\beta} \}$ can converge to different solutions of (1) with $S_{\beta}=S_{\infty}$. This is explained in Sect. 6, which also contains a result addressing the convergence of the entire sequence $\{ \mathbf{u}_{\beta} \}$.

The limit process $\beta\rightarrow\infty$, using different techniques, is studied in [18, 19] for the stationary solutions of neural field equations. It has also been observed [20] for the Wilson–Cowan model that this transition is a subtle matter: Using a steep sigmoid firing rate function instead of the Heaviside mapping can lead to significant changes in a Hopf bifurcation point. ‘*the limiting value of the Hopf depends on the choice of the firing rate function*’.

If one uses a Heaviside firing rate function in (1) the right-hand-sides of these ODEs become discontinuous. A rather general theory for such equations has been developed [21]. In this theory the system of ODEs is replaced by a differential inclusion, in which the right-hand side of the ODE system is substituted by a set-valued function. The construction of this set-valued operator can be accomplished by invoking Filippov regularization/convexification. But this methodology serves a different purpose than the smoothing processes considered in this paper. More specifically, it makes it possible to prove that generalized solutions (Filippov solutions) to the problem exist but do not provide a family of well-posed equations suitable for numerical solution.

Smoothening techniques for discontinuous vector fields, which are similar to the regularization method considered in this paper, have been proposed and analyzed for rather general phase spaces [22–24]. Nevertheless, these studies consider qualitative properties of large classes of problems, whereas we focus on a quantitative analysis of a very special system of ODEs.

For the sake of easy notation, we will sometimes write (1) in the form

3 $$ \begin{aligned} \boldsymbol{\tau} \mathbf{u}'(t) &= - \mathbf{u}(t) + \boldsymbol{\omega} S _{\beta} \bigl[\mathbf{u}(t)- \mathbf{u}_{\theta} \bigr] + \mathbf{q}(t), \quad t \in(0,T], \\ \mathbf{u}(0) &=\mathbf{u}_{\mathrm{init}}, \end{aligned} $$

where

4 $$\begin{aligned} \mathbf{u}(t)&=\mathbf{u}_{\beta} (t) \in \mathbb {R}^{N}, \quad t \in [0,T], \mbox{ see (2)}, \\ \mathbf{q}(t)&= \bigl( q_{1}(t), q_{2}(t), \ldots, q_{N}(t) \bigr) ^{T} \in \mathbb {R}^{N}, \quad t \in (0,T], \\ \mathbf{u}_{\theta}&= ( u_{\theta}, u_{\theta}, \ldots, u_{\theta} ) ^{T} \in \mathbb {R}^{N}, \end{aligned}$$

5 $$\begin{aligned} \mathbf{u}_{\mathrm{init}} &= ( u_{\mathrm{init},1}, u_{ \mathrm{init},2} , \ldots, u_{\mathrm{init},N} ) ^{T} \in \mathbb {R}^{N}, \\ \boldsymbol{\omega} &= [\omega_{i,j}] \in \mathbb {R}^{N \times N}, \\ \boldsymbol{\tau} &= \operatorname{diag}(\tau_{1}, \tau_{2}, \ldots, \tau_{N}) \in \mathbb {R}^{N \times N} \mbox{ is diagonal}, \\ S_{\beta}[\mathbf{x}]&= \bigl(S_{\beta}[x_{1}], \ldots ,S_{\beta}[x_{N}] \bigr)^{T}, \quad\mathbf{x} = (x_{1}, \ldots, x_{N})^{T} \in \mathbb {R}^{N}. \end{aligned}$$

Note that we, for the sake of simplicity, use the same threshold value $u_{\theta}$ for all the units in the network; see (4).

## Assumptions

Throughout this text we use the standard notation

$$ \Vert \mathbf{x} \Vert _{\infty}=\max_{1\leq i\leq N}\vert x_{i} \vert ,\quad\mathbf{x}=(x_{1}, \ldots,x_{N}) \in \mathbb {R}^{N}. $$

Concerning the sequence $\{ S_{\beta} \}$ of finite steepness firing rate functions, we make the following assumption.

### Assumption A

We assume that

- $S_{\beta}$, $\beta\in \mathbb {N}$, is Lipschitz continuous,
- $0 \leq S_{\beta}(x) \leq1$, $x \in \mathbb {R}, \quad \beta\in \mathbb {N}$,
- for every pair of positive numbers $(\epsilon, \delta)$ there exists $Q \in \mathbb {N}$ such that
  6 $$\begin{aligned} \bigl\vert S_{\beta}(x) \bigr\vert &< \epsilon \quad \mbox{for } x < -\delta\mbox{ and } \beta> Q, \end{aligned}$$
  7 $$\begin{aligned} \bigl\vert 1-S_{\beta}(x) \bigr\vert &< \epsilon \quad \mbox{for } x > \delta\mbox{ and } \beta> Q. \end{aligned}$$

There are many continuous sigmoidal functions which approximate the Heaviside step function and satisfy Assumption A. For example,

8 $$\begin{aligned} S(x) &= \frac{1}{2} \bigl(1+\tanh(x) \bigr) , \end{aligned}$$

9 $$\begin{aligned} S_{\beta}[x] &= S(\beta x). \end{aligned}$$

More generally, if $S_{\beta}$ is nondecreasing (for every $\beta\in \mathbb {N}$), a) and b) hold and $\{ S_{\beta} \}$ converges pointwise to the Heaviside function, then Assumption A holds. Also, if Assumption A is satisfied and $\lim_{\beta\rightarrow\infty} S_{\beta}(0) = S_{\infty}(0)=H(0)$, then $\{ S_{\beta} \}$ converges pointwise to the Heaviside function.

We will consider a slightly more general version of the model than (3). More specifically, we allow the source term to depend on the steepness parameter, $\mathbf{q}=\mathbf{q}_{\beta}$, but in such a way that the following assumption holds.

### Assumption B

We assume that $\mathbf{q}_{\beta} (t)$, $t \in[0,T]$, $\beta\in \mathbb {N}\cup\{ \infty\}$ is continuous and that

10 $$\begin{aligned} \sup_{\beta\in \mathbb {N}, t \in[0,T]} \bigl\Vert \mathbf{q}_{\beta} (t) \bigr\Vert _{\infty} &\leq B < \infty, \quad B \in \mathbb {R}, \end{aligned}$$

11 $$\begin{aligned} \lim_{\beta\rightarrow\infty} \mathbf{q}_{\beta} (t)& = \mathbf{q_{\infty}} (t), \quad t \in[0,T], \\ \lim_{\beta\rightarrow\infty} \int_{0}^{t} \mathbf{q}_{\beta}(s)\,ds& = \int_{0}^{t} \mathbf{q_{\infty}}(s)\,ds, \quad t \in[0,T]. \end{aligned}$$

Allowing the external drive to depend on the steepness parameter makes it easier to construct illuminating examples. However, we note that our theory will also hold for the simpler case when **q** does not change as *β* increases.

In this paper we will assume that Assumptions A and B are satisfied.

## Uniformly Bounded and Equicontinuous

In order to apply the Arzelà–Ascoli theorem we must show that $\{ \mathbf{u}_{\beta} \}$ constitutes a family of uniformly bounded and equicontinuous functions. (For the sake of simple notation, we will write $u_{i}$ and $q_{i}$, instead of $u_{\beta,i}$ and $q_{\beta,i}$, for the component functions of $\mathbf{u}_{ \beta}$ and $\mathbf{q}_{\beta}$, respectively.) Multiplying

$$ u'_{i}(s) + \tau_{i}^{-1} u_{i}(s) = \tau_{i}^{-1} \sum _{j=1}^{N} \omega_{i,j} S_{\beta} \bigl[u_{j}(s)-u_{\theta} \bigr] + \tau_{i}^{-1} q_{i}(s) $$

with $e^{s/\tau_{i}}$ yields that

$$ \bigl[ u_{i}(s) e^{s/\tau_{i}} \bigr] ' = e^{ s/\tau_{i}} \tau_{i}^{-1} \sum _{j=1}^{N} \omega_{i,j} S_{\beta} \bigl[u_{j}(s)-u_{\theta} \bigr] + e^{s/\tau_{i}} \tau_{i}^{-1} q_{i}(s) $$

and by integrating

$$ u_{i}(t) e^{t/\tau_{i}} = u_{i}(0) + \int_{0}^{t} e ^{s/\tau_{i}} \tau_{i}^{-1} \sum_{j=1}^{N} \omega_{i,j} S_{\beta} \bigl[u_{j}(s)-u_{\theta} \bigr]\,ds + \int_{0}^{t} e^{ s/\tau_{i}} \tau_{i}^{-1} q_{i}(s)\,ds. $$

Hence, since $S_{\beta}[x] \in[0,1]$ and we assume that $\tau_{i} > 0$ for $i=1,2,\ldots,N$,

$$\begin{aligned} \bigl\vert u_{i}(t) \bigr\vert e^{t/\tau_{i}} &\leq\bigl\vert u_{i}(0) \bigr\vert + \sum_{j=1}^{N} \vert \omega_{i,j} \vert \int_{0}^{t} e^{ s/\tau_{i}} \tau_{i}^{-1} \,ds + \sup_{s \in[0,T]} \bigl\vert q_{i}(s) \bigr\vert \int_{0}^{t} e^{s/\tau_{i}} \tau_{i}^{-1} \,ds \\ &= \bigl\vert u_{i}(0) \bigr\vert + \Biggl( \sum _{j=1}^{N} \vert \omega_{i,j} \vert + \sup_{s \in[0,T]} \bigl\vert q_{i}(s) \bigr\vert \Biggr) \bigl( e^{t/\tau_{i}} - 1 \bigr) \\ &\leq\bigl\vert u_{i}(0) \bigr\vert + \Biggl( \sum _{j=1}^{N} \vert \omega_{i,j} \vert + B \Biggr) \bigl( e^{t/\tau_{i}} - 1 \bigr) ,\quad t \in(0,T], \end{aligned}$$

where the last inequality follows from (10). This implies that

12 $$ \bigl\Vert \mathbf{u}_{\beta}(t) \bigr\Vert _{\infty} \leq\underbrace{\Vert \mathbf{u}_{\mathrm{init}} \Vert _{\infty} + \max_{i} \Biggl( \sum _{j=1}^{N} \vert \omega_{i,j} \vert \Biggr) + B}_{=\widetilde{B}},\quad t \in[0,T]. $$

Since the right-hand side of (12) is independent of *β* and *t* we conclude that the sequence $\{ \mathbf{u}_{ \beta} \}$ is uniformly bounded.

Next, from the model (3), the triangle inequality, the assumption that $S_{\beta}[x] \in[0,1]$ and assumption (10) we find that

$$ \bigl\Vert \boldsymbol{\tau} \mathbf{u}_{\beta}'(t) \bigr\Vert _{\infty} \leq\widetilde{B} + \max_{i} \Biggl( \sum_{j=1}^{N} \vert \omega_{i,j} \vert \Biggr) + B, \quad t \in(0,T], $$

where *B̃* is defined in (12). Since ***τ*** is a diagonal matrix with positive entries on the diagonal, this yields that

$$ \bigl\Vert \mathbf{u}_{\beta}'(t) \bigr\Vert _{\infty} \leq\frac{1}{\min_{i} \{ \tau_{i} \}} \Biggl[ \widetilde {B} + \max _{i} \Biggl( \sum_{j=1}^{N} \vert \omega_{i,j} \vert \Biggr) + B \Biggr] = K, \quad t \in (0,T]. $$

Here the constant *K* is independent of both *β* and $t \in(0,T]$.

Let $i \in\{ 1,2, \ldots, N \}$ and $\beta\in \mathbb {N}$ be arbitrary. Then, for any time instances $t_{1}, t_{2} \in[0,T]$, with $t_{1} < t_{2}$, the mean value theorem implies that there exists $t^{*} \in(t_{1},t_{2})$ such that

$$ u_{i}(t_{2})-u_{i}(t_{1}) = u_{i}' \bigl(t^{*} \bigr) (t_{2}-t_{1}), $$

and hence

$$ \bigl\vert u_{i}(t_{2})-u_{i}(t_{1}) \bigr\vert = \bigl\vert u_{i}' \bigl(t^{*} \bigr) \bigr\vert \bigl\vert (t_{2}-t_{1}) \bigr\vert \leq K \bigl\vert (t_{2}-t_{1}) \bigr\vert . $$

This inequality holds for any $i \in\{ 1,2, \ldots, N \}$, $\beta\in \mathbb {N}$. It therefore follows that

$$ \bigl\Vert \mathbf{u}_{\beta}(t_{2}) - \mathbf{u}_{\beta}(t_{1}) \bigr\Vert _{\infty} \leq K \bigl\vert (t_{2}-t_{1}) \bigr\vert , \quad t_{1}, t_{2} \in[0,T] \mbox{ and } \beta\in \mathbb {N}, $$

from which we conclude that $\{ \mathbf{u}_{\beta} \}$ is a set of equicontinuous functions

The Arzelà–Ascoli theorem now asserts that there is a uniformly convergent subsequence $\{ \mathbf{u}_{\beta_{k}} \}$:

13 $$ \mathbf{v} = \lim_{k \rightarrow\infty} \mathbf {u}_{\beta_{k}}. $$

According to standard ODE theory, $\mathbf{u}_{\beta_{k}}$ is continuous for each $k \in \mathbb {N}$. Hence the uniform convergence implies that **v** is also continuous.

### Threshold Terminology

As we will see in subsequent sections it depends on **v**’s threshold properties whether we can prove that **v** actually solves the limit problem with a Heaviside firing rate function. The following concepts turn out to be useful.

For a vector-valued function $\mathbf{z} = (z_{1},z_{2},\ldots,z_{N})^{T}: [0,T] \rightarrow \mathbb {R}^{N}$ we define

14 $$ m(s;\mathbf{z}) = \min_{j \in\{1,2,\ldots, N\}} \bigl\vert z_{j}(s)-u_{\theta} \bigr\vert , \quad s \in[0,T]. $$

#### Definition

Threshold simple

A measurable vector-valued function $\mathbf{z}:[0,T] \rightarrow \mathbb {R}^{N}$ is threshold simple if the Lebesgue measure of the set

15 $$ Z(\mathbf{z}) = \bigl\{ s \in[0,T] \mid m(s;\mathbf {z})=0 \bigr\} $$

is zero, i.e. $\vert Z(\mathbf{z}) \vert =0$.

#### Definition

Extra threshold simple

A measurable vector-valued function $\mathbf{z}:[0, T] \rightarrow \mathbb {R}^{N}$ is extra threshold simple if there exist open intervals

$$ I_{l} = (a_{l}, a_{l+1}), \quad l=1,2,\ldots, L, $$

such that

$$\begin{aligned} a_{1}&=0, \qquad a_{L+1} = T, \\ m(s;\mathbf{z}) &\neq0 \quad\forall s \in\bigcup _{l=1}^{L} I_{l}. \end{aligned}$$

In words, **z** is extra threshold simple if there is a finite number of threshold crossings on the time interval $[0,T]$.

## The Limit of the Subsequence

### Preparations

We will prove that the limit **v** in (13) solves the integral form of (3) with $S_{\infty} = H$, the Heaviside function, provided that **v** is threshold simple. The inhomogeneous nonlinear Volterra equation associated with (3) reads

16 $$\begin{aligned} \boldsymbol{\tau} \mathbf{u}_{\beta_{k}}(t) - \boldsymbol{\tau} \mathbf{u} _{\mathrm{init}}&= - \int_{0}^{t} \mathbf{u}_{\beta_{k}}(s)\,ds \\ & \quad {}+ \int_{0}^{t} \boldsymbol{\omega} S_{\beta_{k}} \bigl[ \mathbf{u}_{\beta_{k}}(s)- \mathbf{u}_{\theta} \bigr]\,ds \\ & \quad {}+ \int_{0}^{t} \mathbf{q}_{\beta_{k}}(s)\,ds, \quad t \in[0,T], \end{aligned}$$

where

$$ \int_{0}^{t} \mathbf{u}_{\beta_{k}}(s)\,ds = \biggl( \int_{0}^{t} u_{\beta_{k},1}(s)\,ds, \int_{0}^{t} u_{\beta_{k},2}(s)\,ds, \ldots, \int_{0}^{t} u_{\beta_{k},N}(s)\,ds \biggr) ^{T}, $$

etc.; see also (2) and (5). Note that we consider the equations satisfied by the subsequence $\{ \mathbf{u}_{\beta_{k}} \}$, see (13). We will analyze the convergence of the entire sequence in Sect. 6.

The uniform convergence of $\{ \mathbf{u}_{\beta_{k}} \}$ to **v** implies that the left-hand-side and the first integral on the right-hand side of (16) converge to $\boldsymbol{\tau} \mathbf{v}(t) - \boldsymbol{\tau} \mathbf {u}_{\mathrm{init}}$ and $- \int_{0}^{t} \mathbf{v}(s)\,ds$, respectively, as $k \rightarrow\infty$. Also, due to assumption (11), the third integral on the right-hand side does not require any extra attention. We will thus focus on the second integral on the right-hand side of (16).

For $t \in[0,T]$ and $\delta> 0$, define the sets

17 $$\begin{aligned} p(\delta;t) &= \bigl\{ s \in[0,t] \mid m(s;\mathbf{v}) > \delta\bigr\} , \\ r(\delta;t)& = [0,t] \setminus p(\delta;t), \end{aligned}$$

where $m(s;\mathbf{v})$ is defined in (14) and **v** is the limit in (13). Since **v** is continuous it follows that $m(s;\mathbf{v})$, $s \in[0,T]$, is continuous. Hence, the sets $p(\delta;t) $ and $r(\delta;t)$ are Lebesgue measurable. We note that, provided that $\delta>0$ is small, the set $r(\delta;t)$ contains the times where at least one of the components of **v** is close to the threshold value $u_{\theta}$ for firing. The following lemma turns out to be crucial for our analysis of the second integral on the right-hand side of (16).

#### Lemma 4.1

*If the limit function* **v** *in* (13) *is threshold simple*, *then*

18 $$ \lim_{\delta\rightarrow0^{+}} \bigl\vert r(\delta;t) \bigr\vert = 0, \quad t \in[0,T], $$

*where* $\vert r(\delta;t) \vert $ *denotes the Lebesgue measure of the set* $r(\delta;t)$.

#### Proof

Since **v** is the uniform limit of a sequence of continuous functions, **v** is continuous and hence measurable. If **v** is threshold simple, then

19 $$ \bigl\vert Z(\mathbf{v}) \bigr\vert =0, $$

see (15).

Let $t \in[0,T]$ be arbitrary. Assume that

$$ \lim_{\delta\rightarrow0^{+}} \bigl\vert r(\delta;t) \bigr\vert \neq0, $$

or that this limit does not exist. Then $\exists \tilde{\epsilon} > 0$ such that there is a sequence $\{ \delta_{n} \}$ satisfying

$$\begin{aligned} 0 &< \delta_{n+1} < \delta_{n} \quad\forall n \in \mathbb {N}, \\ \lim_{n \rightarrow\infty} \delta_{n} &= 0, \\ \bigl\vert r(\delta_{n};t) \bigr\vert &> \tilde{\epsilon} \quad\forall n \in \mathbb {N}. \end{aligned}$$

By construction,

$$ r(\delta_{1};t) \supset r(\delta_{2};t) \supset\cdots \supset r(\delta_{n};t) \supset\ldots, $$

and $\vert r(\delta_{1};t) \vert \leq T < \infty$. Hence,

$$ \Biggl\vert \bigcap_{n=1}^{\infty} r(\delta _{n};t) \Biggr\vert = \lim_{n \rightarrow\infty} \bigl\vert r( \delta_{n};t) \bigr\vert \geq\tilde{\epsilon} > 0, $$

see, e.g., [13] (page 62). Since the sequence $\{ \vert r(\delta_{n};t) \vert \}$ is nonincreasing and bounded below, $\lim _{n \rightarrow\infty} \vert r(\delta_{n};t) \vert $ exists.

Next,

$$ s \in\bigcap_{n=1}^{\infty} r(\delta _{n};t)\quad\Rightarrow\quad m(s;\mathbf{v}) \leq\delta _{n} \quad\forall n\quad\Rightarrow\quad m(s;\mathbf{v})=0\quad \Rightarrow\quad s \in Z(\mathbf{v}), $$

i.e.

$$ \bigcap_{n=1}^{\infty} r(\delta _{n};t) \subset Z(\mathbf{v}). $$

Hence,

$$ \bigl\vert Z(\mathbf{v}) \bigr\vert \geq\Biggl\vert \bigcap _{n=1}^{\infty} r(\delta_{n};t) \Biggr\vert \geq\tilde{\epsilon} > 0, $$

which contradicts (19). □

### Convergence of the Integral

#### Lemma 4.2

*If the limit* **v** *in* (13) *is threshold simple*, *then*

20 $$ \lim_{k \rightarrow\infty} \int_{0}^{t} \boldsymbol{\omega} S_{\beta _{k}} \bigl[ \mathbf{u}_{\beta_{k}}(s)-\mathbf{u}_{\theta} \bigr]\,ds = \int_{0}^{t} \boldsymbol{\omega} S_{\infty} \bigl[\mathbf{v}(s)- \mathbf{u}_{\theta} \bigr]\,ds, \quad t \in[0,T]. $$

#### Proof

Let $t \in[0,T]$ and $\tilde{\epsilon} > 0$ be arbitrary and define

$$ C= \max_{i \in\{1,2,\ldots,N\}} \Biggl( \sum_{j=1}^{N} \vert \omega_{i,j} \vert \Biggr) . $$

From (18) we know that there exists $\Delta> 0$ such that

21 $$ \bigl\vert r(2\delta;t) \bigr\vert < \frac{\tilde {\epsilon}}{2C}, \quad0 < \delta< \Delta. $$

Choose a *δ* which satisfies $0 < \delta< \Delta$. By part (c) of Assumption A, for this *δ* and

22 $$ \epsilon=\frac{\tilde{\epsilon}}{2TC}, $$

there exists $Q \in \mathbb {N}$ such that (6) and (7) hold.

Recall that $\beta_{1}, \beta_{2}, \ldots, \beta_{k}, \ldots$ are the values for the steepness parameter associated with the convergent subsequence $\{ \mathbf{u}_{\beta_{k}} \}$ in (13). By the uniform convergence of $\{ \mathbf{u}_{\beta_{k}} \}$ to **v**, there is a $K \in \mathbb {N}$ so that

23 $$\begin{aligned} \beta_{K} &> Q, \end{aligned}$$

24 $$\begin{aligned} \sup_{s \in[0,T]} \bigl\Vert \mathbf{u}_{\beta_{k}}(s) - \mathbf{v}(s) \bigr\Vert _{\infty} &< \delta, \quad k > K. \end{aligned}$$

From the definition of the set $p(2 \delta;t)$, see (17) and (14),

25 $$ m(s; \mathbf{v}) = \min_{ j \in\{1,2,\ldots, N \}} \bigl\vert v_{j}(s)-u_{\theta} \bigr\vert > 2 \delta> \delta, \quad s \in p(2 \delta;t), $$

and from (24) and the triangle inequality it follows that

26 $$ \min_{ j \in\{1,2,\ldots, N \}} \bigl\vert u_{\beta _{k},j}(s)-u_{\theta} \bigr\vert > \delta, \quad s \in p(2 \delta;t)\mbox{ and } k > K. $$

From (24)–(26) we find that

$$ \bigl(v_{j}(s)-u_{\theta} \bigr) \cdot\bigl(u_{\beta _{k},j}(s)-u_{\theta} \bigr) > 0, \quad s \in p(2 \delta;t),j \in\{1,2,\ldots, N \}, k > K. $$

Also, because of the properties of the Heaviside function,

$$ S_{\infty} \bigl(v_{j}(s)-u_{\theta} \bigr) = \textstyle\begin{cases} 1, & v_{j}(s)-u_{\theta} \geq\delta, \\ 0 & v_{j}(s)-u_{\theta} \leq-\delta, \end{cases} $$

$j \in\{1,2,\ldots, N \}$. Consequently, due to (23) and part (c) of Assumption A, see (6) and (7), we find that

$$ \begin{aligned} &\bigl\vert S_{\beta_{k}} \bigl[u_{\beta_{k},j}(s)-u_{\theta} \bigr] - S_{\infty} \bigl[v_{j}(s)-u_{\theta} \bigr] \bigr\vert < \epsilon, \\ &\quad s \in p(2 \delta;t), j \in\{ 1,2, \ldots, N \}, k > K. \end{aligned} $$

Hence,

$$\begin{aligned} & \biggl\Vert \int_{0}^{t} \boldsymbol{\omega} \bigl\{ S_{\beta_{k}} \bigl[\mathbf{u} _{\beta_{k}}(s)-\mathbf{u}_{\theta} \bigr] - S_{\infty} \bigl[\mathbf{v}(s)- \mathbf{u}_{\theta} \bigr] \bigr\} \,ds \biggr\Vert _{\infty} \\ & \quad= \biggl\Vert \int_{p(2\delta;t) \cup r(2\delta;t)} \boldsymbol{\omega} \bigl\{ S_{\beta_{k}} \bigl[ \mathbf{u}_{\beta_{k}}(s)- \mathbf{u}_{\theta} \bigr] - S_{\infty} \bigl[\mathbf{v}(s)- \mathbf{u}_{\theta} \bigr] \bigr\} \,ds \biggr\Vert _{\infty} \\ & \quad\leq\biggl\Vert \int_{p(2\delta;t)} \boldsymbol{\omega} \bigl\{ S _{\beta_{k}} \bigl[ \mathbf{u}_{\beta_{k}}(s)-\mathbf{u}_{\theta} \bigr] - S_{ \infty} \bigl[\mathbf{v}(s)-\mathbf{u}_{\theta} \bigr] \bigr\} \,ds \biggr\Vert _{ \infty} \\ & \quad\quad{} + \biggl\Vert \int_{r(2\delta;t)} \boldsymbol{\omega} \bigl\{ S _{\beta_{k}} \bigl[ \mathbf{u}_{\beta_{k}}(s)-\mathbf{u}_{\theta} \bigr] - S_{ \infty} \bigl[\mathbf{v}(s)-\mathbf{u}_{\theta} \bigr] \bigr\} \,ds \biggr\Vert _{ \infty} \\ & \quad\leq\epsilon\bigl\vert p(2\delta;t) \bigr\vert \max _{i \in\{1,2,\ldots,N \}} \Biggl( \sum_{j=1}^{N} \vert \omega_{i,j} \vert \Biggr) \\ & \quad\quad{} + \bigl\vert r(2\delta;t) \bigr\vert \max _{i \in\{1,2,\ldots,N\}} \Biggl( \sum_{j=1}^{N} \vert \omega_{i,j} \vert \Biggr) \\ & \quad\leq\frac{\tilde{\epsilon}}{2TC} T \max_{i \in\{1,2,\ldots ,N\}} \Biggl( \sum _{j=1}^{N} \vert \omega_{i,j} \vert \Biggr) \\ &\quad\quad{} +\frac{\tilde{\epsilon}}{2C} \max_{i \in\{1,2,\ldots ,N\}} \Biggl( \sum _{j=1}^{N} \vert \omega_{i,j} \vert \Biggr) \\ & \quad< \tilde{\epsilon} \end{aligned}$$

for all $k > K$, where the second last inequality follows from (22), the fact that $\vert p(2\delta;t) \vert \leq T$ for $t \in[0,T]$ and (21). Since $\tilde{\epsilon} > 0$ and $t \in[0,T]$ were arbitrary, we conclude that (20) must hold. □

### Limit Problem

By employing the uniform convergence (13), the convergence of the integral (20) and assumption (11), we conclude from (16) that the limit function **v** satisfies

27 $$\begin{aligned} \boldsymbol{\tau} \mathbf{v}(t) - \boldsymbol{\tau} \mathbf{u}_{ \mathrm{init}} &= {-} \int_{0}^{t} \mathbf{v}(s)\,ds+ \int_{0}^{t} \boldsymbol{\omega} S_{\infty} \bigl[ \mathbf{v}(s)- \mathbf{u}_{\theta} \bigr]\,ds \\ &\quad {} + \int_{0}^{t} \mathbf{q}_{\infty}(s)\,ds, \quad t \in[0,T], \end{aligned}$$

provided that **v** is threshold simple. Recall that **v** is continuous. Consequently, if **v** is extra threshold simple, then it follows from the fundamental theorem of calculus that **v** also satisfies the ODEs, except at time instances when one or more of the component functions equal the threshold value for firing:

28 $$ \begin{aligned} \boldsymbol{\tau} \mathbf{v}'(t)&= - \mathbf{v}(t) + \boldsymbol{\omega} S _{\infty} \bigl[\mathbf{v}(t)- \mathbf{u}_{\theta} \bigr] + \mathbf{q}_{\infty}(t), \quad t \in(0,T] \setminus Z(\mathbf{v}), \\ \mathbf{v}(0)&= \mathbf{u}_{\mathrm{init}}, \end{aligned} $$

where $Z(\mathbf{v})$ is defined in (15).

The existence of a solution matter for point neuron models with a Heaviside firing rate function is summarized in the following theorem.

#### Theorem 4.3

*If the limit* **v** *in* (13) *is threshold simple*, *then* **v** *solves* (27). *In the case that* **v** *is extra threshold simple* **v** *also satisfies* (28).

In [25] the existence issue for neural field equations with a Heaviside activation function is studied but the analysis is different because a continuum model is considered. We would also like to mention that Theorem 4.3 cannot be regarded as a simple consequence of Carathéodory’s existence theorem [21, 26, 27] because the right-hand-side of (28) is discontinuous with respect to **v**.

## Uniqueness

If $\beta< \infty$, then standard ODE theory [15–17] implies that (3) has a unique solution. Unfortunately, as will be demonstrated below, this desirable property is not necessarily inherited by the infinite steepness limit problem.

We will first explain why the uniqueness question is a subtle issue for point neuron models with a Heaviside firing rate function. Thereafter, additional requirements are introduced which ensure the uniqueness of an extra threshold simple solution.

### Example: Several Solutions

Let us study the problem

29 $$ \begin{aligned} v'(t) &= -v(t) + \omega S_{\infty} \bigl[v(t)-u_{\theta} \bigr], \quad t \in(0,T], \\ v(0) &= u_{\theta}, \end{aligned} $$

where we assume that

$$ \omega>u_{\theta} \geq0. $$

Note that the ODE in (29) is not required to hold for $t=0$. Consider the functions

30 $$\begin{aligned} v_{1}(t) &= \omega+ (u_{\theta}-\omega)e^{-t} = u_{\theta} e^{-t} + \bigl(1-e^{-t} \bigr) \omega, \end{aligned}$$

31 $$\begin{aligned} v_{2}(t)& = u_{\theta} e^{-t}. \end{aligned}$$

Since

$$\begin{aligned} v_{1}(t) &> u_{\theta} e^{-t} + \bigl(1-e^{-t} \bigr) u_{\theta} = u_{\theta}, \quad t \in(0,T], \\ v_{2}(t) &< u_{\theta}, \quad t \in(0,T], \end{aligned}$$

it follows that both $v_{1}$ and $v_{2}$ solves (29).

Furthermore, with

$$\begin{aligned} \omega&=2 u_{\theta}, \\ S_{\infty}(0)&=\frac{1}{2}, \end{aligned}$$

we actually obtain a third solution of (29). More specifically, the stationary solution

32 $$ v_{3}(t)=u_{\theta}, \quad t \in[0,T]. $$

We conclude that models with a Heaviside firing rate function can have several solutions – such problems can thus become ill-posed. (In [9] we showed that the *initial-condition-to-solution* map is not necessarily continuous for such problems and that the error amplification ratio can become very large in the steep but Lipschitz continuous firing rate regime.) Note that switching to the integral form (27) will not resolve the lack of uniqueness issue for the toy example considered in this subsection.

We also remark that

- If we define $S_{\infty}(0)=1/2$, then neither $v_{1}$ nor $v_{2}$ satisfies the ODE in (29) for $t=0$. (In the case $\omega=2 u_{\theta}$, $v_{3}$ satisfies the ODE in (29) for $t=0$.)
- If we define $S_{\infty}(0)=1$, then $v_{1}$, but not $v_{2}$, satisfies the ODE in (29) also for $t=0$.
- If we define $S_{\infty}(0)=0$, then $v_{2}$, but not $v_{1}$, satisfies the ODE in (29) also for $t=0$.

### Enforcing Uniqueness

In order to enforce uniqueness we need to impose further restrictions. It turns out that it is sufficient to require that the derivative is continuous from the right and that the ODEs also must be satisfied whenever one, or more, of the component functions equals the threshold value for firing

33 $$ \begin{aligned} \boldsymbol{\tau} \mathbf{v}'(t)&= - \mathbf{v}(t) + \boldsymbol{\omega} S _{\infty} \bigl[\mathbf{v}(t)- \mathbf{u}_{\theta} \bigr] + \mathbf{q}_{\infty}(t), \quad t \in [0,T], \\ \mathbf{v}(0) &=\mathbf{u}_{\mathrm{init}}. \end{aligned} $$

Note that the ODEs in (33) also must be satisfied for $t=0$, in case one of the components of $\mathbf{u}_{ \mathrm{init}}$ equals $u_{\theta}$.

#### Definition 1

Right smooth

A vector-valued function $\mathbf{z}:[0,T] \rightarrow \mathbb {R}^{N}$ is right smooth if $\mathbf{z}'$ is continuous from the right for all $t \in[0,T)$.

#### Theorem 5.1

*The initial value problem* (33) *can have at most one solution which is both extra threshold simple and right smooth*.

#### Proof

Let **v** and $\tilde{\mathbf{v}}$ be two solutions of (33) which are both right smooth and extra threshold simple:

$$\begin{aligned}{} [0,T] &= \bigcup_{l=1}^{L} \bar{I}_{l}, \\ m(s;\mathbf{v}) &\neq0 \quad\forall s \in\bigcup_{l=1}^{L} I_{l}, \end{aligned}$$

and

$$\begin{aligned}{} [0,T] &= \bigcup_{l=1}^{\tilde{L}} \bar{ \tilde{I}}_{l}, \\ m(s;\tilde{\mathbf{v}}) &\neq0 \quad\forall s \in\bigcup _{l=1} ^{\tilde{L}} \tilde{I}_{l}, \end{aligned}$$

where $I_{1}, I_{2}, \ldots, I_{L}$ and $\tilde{I}_{1}, \tilde{I} _{2}, \ldots, \tilde{I}_{\tilde{L}}$ are disjoint open intervals; see (14) and the definition of extra threshold simple in Sect. 3.1.

Then there exist disjoint open intervals $\hat{I}_{1}, \hat{I}_{2}, \ldots, \hat{I}_{\hat{L}}$ such that

34 $$\begin{aligned}{} [0,T]& = \bigcup_{l=1}^{\hat{L}} \bar{\hat{I}}_{l}, \\ m(s;\mathbf{v})& \neq0 \quad\mbox{and} \quad m(s;\tilde{\mathbf{v}}) \neq0 \quad\forall s \in\bigcup_{l=1}^{\hat{L}} \hat{I}_{l}. \end{aligned}$$

Let us focus on one of these intervals, $\hat{I}_{l} = (a_{l},a_{l+1})$. Define

$$ \mathbf{d}=\mathbf{v}-\tilde{\mathbf{v}} $$

and assume that

35 $$ \mathbf{v}(a_{l}) = \tilde{\mathbf{v}}(a_{l}), $$

which obviously holds for $l=1$. Then

36 $$\begin{aligned} \boldsymbol{\tau} \mathbf{d}'(t) &= - \mathbf{d}(t) + \boldsymbol{\omega} \boldsymbol{\gamma}(t),\quad t \in[a_{l},a_{l+1}], \end{aligned}$$

37 $$\begin{aligned} \mathbf{d}(a_{l}) &=\mathbf{0}, \end{aligned}$$

where

$$ \boldsymbol{\gamma}(t) = S_{\infty} \bigl[\mathbf{v}(t)-\mathbf {u}_{\theta} \bigr] - S_{\infty} \bigl[\tilde{\mathbf{v}}(t)-\mathbf{u}_{\theta} \bigr], \quad t \in[a_{l},a_{l+1}]. $$

Note that, due to (34), $\boldsymbol{\gamma}(t)$ equals a constant vector **c**, with components $-1,0$ or 1, except possibly at $t=a_{l}, a_{l+1}$:

38 $$ \boldsymbol{\gamma}(t) = \mathbf{c}, \quad t \in (a_{l},a_{l+1}). $$

Furthermore, from (35) we find that

39 $$ \boldsymbol{\gamma}(a_{l}) = \mathbf{0}. $$

Putting $t=a_{l}$ in (36) and invoking (37) and (39) yield

$$ \mathbf{d}'(a_{l}) = \mathbf{0}, $$

and from the right continuity of $\mathbf{d}'$ and **d**, (36), (37) and (38) we find that

$$ \begin{aligned} \mathbf{0} &= \boldsymbol{\tau} \mathbf{d}'(a_{l}) \\ &= \lim _{t \rightarrow a_{l}^{+}} \boldsymbol{\tau} \mathbf{d}'(t) \\ &= \lim _{t \rightarrow a_{l}^{+}} \bigl[- \mathbf{d}(t) + \boldsymbol{\omega} \boldsymbol{\gamma}(t) \bigr] \\ &= \boldsymbol{\omega} \mathbf{c}. \end{aligned} $$

Since $\boldsymbol{\omega} \boldsymbol{\gamma}(t) = \boldsymbol{\omega} \mathbf{c} = \mathbf{0}$, $t \in(a_{l}, a_{l+1})$, and $\boldsymbol{\omega} \boldsymbol{\gamma}(a_{l}) =\mathbf{0} $, see (39), we conclude from (36)–(37) that **d** satisfies

$$\begin{aligned} \boldsymbol{\tau} \mathbf{d}'(t)& = - \mathbf{d}(t), \quad t \in [a_{l},a _{l+1}), \\ \mathbf{d}(a_{l}) &=\mathbf{0}, \end{aligned}$$

which has the unique solution $\mathbf{d}(t) = \mathbf{0}$, $t \in[a_{l},a_{l+1})$. Both $\mathbf{v}(t)$ and $\tilde{\mathbf{v}}(t)$ are differentiable on $[0,T]$ and hence continuous. It follows that, by employing the continuity of **v** and $\tilde{\mathbf{v}}$ at time $t=a_{l+1}$,

$$ \mathbf{v}(t) = \tilde{\mathbf{v}} (t), \quad t \in[a_{l},a_{l+1}]. $$

Since $\mathbf{v}(a_{l+1}) = \tilde{\mathbf{v}} (a_{l+1})$ we can repeat the argument on the next interval $[a_{l+1},a_{l+2}]$. It follows by induction that $\mathbf{v}(t) = \tilde{\mathbf{v}} (t), t \in[0,T]$. □

We would like to comment the findings presented in the bullet-points at the end of Sect. 5.1 in view of Theorem 5.1: In order to enforce uniqueness for the solution of (29) we can require that the ODE in (29) also should be satisfied for $t=0$. Nevertheless, this might force us to define $S_{\infty}(0) \neq \frac{1}{2}$, which differs from the standard definition of the Heaviside function *H*.

More generally, if one has accomplished to compute an extra threshold simple and right smooth function **v** which satisfies (27), one can attempt to redefine $S_{\infty}[\mathbf{v}(t)- \mathbf{u}_{\theta}]$, $t \in\{a_{1}, a_{2}, \ldots, a_{L+1} \}$, such that (33) holds and **v** is the only solution to this problem. This may imply that $S_{\infty}[\mathbf{v}(t)- \mathbf{u}_{\theta}]$ cannot be generated by using the composition $H \circ[\mathbf{v}(t)-\mathbf{u}_{\theta}]$. Instead one must determine $z_{j,k}=S_{\infty} [v_{j}(a_{k})-u_{\theta}]$, $j=1, 2, \ldots, N$, $k = 1, 2, \ldots, L+1$. More precisely, for each $k \in\{1, 2, \ldots, L+1 \}$ one gets a linear system of algebraic equations

$$ \tau_{i} v'_{i}(a_{k}) = -v_{i}(a_{k}) + \sum_{j=1}^{N} \omega_{i,j} z _{j,k} + q_{\infty,i}(a_{k}),\quad i=1,2,\ldots,N, $$

which will have a unique solution $(z_{1,k}, z_{2,k}, \ldots, z_{N,k})^{T}$ if the connectivity matrix $\boldsymbol{\omega} = [ \omega_{i,j}]$ is nonsingular. (In this paragraph, $\{0=a_{1}, a_{2}, \ldots, a_{L+1}=T \}$ are the time instances employed in the definition of extra threshold simple; see Sect. 3.1.)

## Convergence of the Entire Sequence

We have seen that point neuron models with a Heaviside firing rate function can have several solutions. One therefore might wonder if different subsequences of $\{ \mathbf{u}_{\beta} \}$ can converge to different solutions of the limit problem. In this section we present an example which shows that this can happen, even though the involved sigmoid functions satisfy Assumption A.

### Example: Different Subsequences Can Converge to Different Solutions

Let us again consider the initial value problem (29), which we discussed in Sect. 5.1. A finite steepness approximation of this problem, using the notation $u(t) =u_{\beta} (t)$, reads:

40 $$ \begin{aligned} &u'(t) = -u(t) + \omega \bar{S}_{\beta} \bigl[u(t)-u_{\theta} \bigr], \quad t \in(0,T], \\ &u(0) = u_{\theta}, \end{aligned} $$

where

$$ \bar{S}_{\beta}[x] = S_{\beta} \biggl[ x+\frac{(-1)^{\beta}}{2 \beta} \biggr] ,\quad \beta\in \mathbb {N}, $$

and $S_{\beta}$ is, e.g., either the hyperbolic tangent sigmoid function (8)–(9) or

41 $$ S_{\beta} (x)= \textstyle\begin{cases} 1,& x > \frac{1}{\beta}, \\ \frac{1}{2}+\frac{1}{2} \beta x ,& x \in [ -\frac{1}{\beta}, \frac{1}{ \beta} ] , \\ 0, & x < -\frac{1}{\beta}. \end{cases} $$

Note that $\{ \bar{S}_{\beta} \}$ converges pointwise, except for $x=0$, to the Heaviside function *H* as $\beta\rightarrow\infty$. In fact, $\{ \bar{S}_{\beta} \}$ satisfies Assumption A.

We consider the case $\omega=2 u_{\theta}$. Therefore (29) has three solutions $v_{1}, v_{2}$ and $v_{3}$, see (30), (31) and (32) in Sect. 5.1. Note that

$$ u(t) =u_{\beta} (t) $$

has the property

**Fig. 1:**
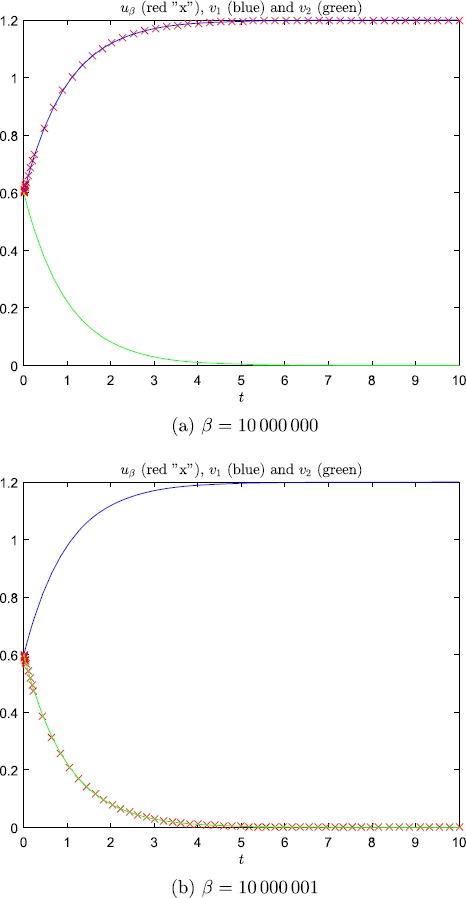
Numerical solutions of (40) computed with Matlab’s ode45 software. In these simulations we used $u_{\theta}=0.6$ and $\omega=1.2$. The functions $v_{1}$ and $v_{2}$, see (30) and (31), are the solutions of the associated limit problem (29)

- $u'_{\beta}(0) > c$ if *β* is even,
- $u'_{\beta}(0) < -c$ if *β* is odd,

where $c>0$ is a constant which is independent of *β*. It therefore follows that

$$\begin{aligned} \lim_{k \rightarrow\infty} u_{2k} &= v_{1}, \\ \lim_{k \rightarrow\infty} u_{2k+1} &= v_{2}, \end{aligned}$$

and no subsequence converges to the third solution $v_{3}$. Figure 1 shows numerical solutions of (40) with steepness parameter $\beta= 10{,}000{,}000, 10{,}000{,}001$, using the firing rate function (41) to define $\bar{S}_{\beta}$. (If one instead employs (8)–(9) in the implementation of $\bar{S}_{\beta}$, the plots, which are not included, are virtually unchanged.)

We would like to mention that we have not been able to construct an example of this kind for Lipschitz continuous firing rate functions which converge pointwise to the Heaviside function also for $x=0$.

### Entire Sequence

We have seen that almost everywhere convergence of the sequence of firing rate functions to the Heaviside limit is not sufficient to guarantee that the entire sequence $\{ u_{\beta} \}$ converges to the same solution of the limit problem. Nevertheless, one has the following result.

#### Theorem 6.1

*Let* **v** *be the limit function in* (13). *If the limit of every convergent subsequence of* $\{ \mathbf{u}_{\beta} \}$ *is extra threshold simple*, *right smooth and satisfies* (33), *then the entire sequence* $\{ \mathbf{u}_{\beta} \}$ *converges uniformly to* **v**.

#### Proof

Suppose that the entire sequence $\{ \mathbf{u}_{\beta} \}$ does not converge uniformly to **v**. Then there is an $\epsilon> 0$ such that, for every positive integer *M*, there must exist $\mathbf{u}_{\beta_{l}}$, $\beta_{l} > M$, satisfying

42 $$ \sup_{t \in[0,T]} \bigl\Vert \mathbf{u}_{\beta_{l}}(t) -\mathbf{v}(t) \bigr\Vert _{ \infty} > \epsilon. $$

Thus, the subsequence $\{ \mathbf{u}_{\beta_{l}} \}$ does not converge uniformly to **v**, but constitutes a set of uniformly bounded and equicontinuous functions, see Sect. 3. According to the Arzelà–Ascoli theorem, $\{ \mathbf{u}_{\beta_{l}} \}$ therefore possesses a uniformly convergent subsequence $\{ \mathbf{u}_{\beta _{l_{n}}} \}$,

$$ \lim_{n \rightarrow\infty} \mathbf{u}_{\beta_{l_{n}}} = \tilde{\mathbf{v}}. $$

Due to (42),

43 $$ \tilde{\mathbf{v}} \neq\mathbf{v}. $$

On the other hand, both **v** and $\tilde{\mathbf{v}}$ are limits of subsequences of $\{ \mathbf{u}_{\beta} \}$ and are by assumption extra threshold simple, right smooth, and they satisfy (33). Hence, Theorem 5.1 implies that $\tilde{\mathbf{v}} = \mathbf{v}$, which contradicts (43). We conclude that the entire sequence $\{ \mathbf{u}_{\beta} \}$ must converge uniformly to **v**. □

## Example: Threshold Advanced Limits

We will now show that threshold advanced limits, i.e. limits which are not threshold simple, may possess some peculiar properties. More precisely, such limits can potentially occur in (13). They do not necessarily satisfy the limit problem obtained by using a Heaviside firing rate function.

With source terms which do not depend on the steepness parameter *β* we have not managed to construct an example with a threshold advanced limit **v**. If we allow $\mathbf{q}=\mathbf{q}_{ \beta}$, this can, however, be accomplished as follows. Let

$$ z_{\beta}(t) = \frac{1}{\beta} S_{\beta} \biggl[- \frac{1}{\beta} + 2t \biggr] + u_{\theta}, \quad\beta= 1,2,\ldots, $$

where we, for the sake of simplicity, work with the firing rate function (41). Then

$$\begin{aligned} z_{\beta}(0) &= \frac{1}{\beta} S_{\beta} \biggl[- \frac{1}{\beta} \biggr] + u _{\theta} = u_{\theta}, \\ z_{\beta}(t) &= \textstyle\begin{cases} t + u_{\theta}, & t \in[0,\frac{1}{\beta}), \\ \frac{1}{\beta} + u_{\theta}, & t \geq\frac{1}{\beta}, \end{cases}\displaystyle \\ z'_{\beta}(t)& = \textstyle\begin{cases} 1, & t \in[0,\frac{1}{\beta}), \\ 0, & t > \frac{1}{\beta}, \end{cases}\displaystyle \\ S_{\beta} \bigl[z_{\beta}(t)- u_{\theta} \bigr]&= \textstyle\begin{cases} \frac{1}{2}+\frac{1}{2} \beta t, & t \in[0,\frac{1}{\beta}), \\ 1, & t \geq\frac{1}{\beta}, \end{cases}\displaystyle \end{aligned}$$

and we find that

$$ u_{\beta}(t) = z_{\beta}(t) $$

solves

$$\begin{aligned} u_{\beta}(t) - u_{\theta} =& - \int_{0}^{t} u_{\beta}(s)\,ds \\ & + \int_{0}^{t} \omega S_{\beta} \bigl[u_{\beta}(s)-u_{\theta} \bigr]\,ds \\ & + \int_{0}^{t} q_{\beta}(s)\,ds, \quad t \in [0,T], \end{aligned}$$

where

44 $$\begin{aligned} q_{\beta}(t) &= z'_{\beta}(t)+z_{\beta}(t)- \omega S_{\beta} \bigl[z_{ \beta}(s)-u_{\theta} \bigr] \\ &= \textstyle\begin{cases} 1+t+u_{\theta}-\omega(\frac{1}{2}+\frac{1}{2} \beta t), & t \in [0,\frac{1}{ \beta}), \\ \frac{1}{\beta}+u_{\theta}-\omega, & t > \frac{1}{\beta}. \end{cases}\displaystyle \end{aligned}$$

It follows that

$$ q_{\infty}(t) = \textstyle\begin{cases} 1+u_{\theta} - \omega, & t=0, \\ u_{\theta} - \omega, & t>0, \end{cases} $$

and since, for any $\beta\in \mathbb {N}$,

$$ \bigl\vert q_{\beta}(t) \bigr\vert \leq1+\frac{1}{\beta}+\vert u_{\theta} \vert +\vert \omega \vert < 2 + \vert u_{\theta} \vert +\vert \omega \vert , \quad t \neq\frac{1}{\beta}, $$

we conclude that

$$ \lim_{\beta\rightarrow\infty} \int_{0}^{t} q_{\beta}(s)\,ds = \int_{0}^{t} q_{\infty}(s)\,ds , \quad t \in [0,T]. $$

Note that

$$ u_{\beta} (t) \longrightarrow\bar{v}(t)=u_{\theta}, \quad \mbox{uniformly, as } \beta\rightarrow\infty, $$

but $\bar{v}(t)=u_{\theta}$ does not solve the limit problem

$$\begin{aligned} v(t) - u_{\theta} &=- \int_{0}^{t} v(s)\,ds \\ & \quad{}+ \int_{0}^{t} \omega S_{\infty} \bigl[v(s)-u_{\theta} \bigr]\,ds \\ &\quad{}+ \int_{0}^{t} q_{\infty}(s)\,ds, \quad t \in [0,T], \end{aligned}$$

because

$$\begin{aligned} & {-} \int_{0}^{t} \bar{v}(s)\,ds + \int_{0}^{t} \omega S_{\infty} \bigl[ \bar{v}(s)-u_{\theta} \bigr]\,ds + \int_{0}^{t} q_{\infty}(s)\,ds \\ &\quad= -t u_{\theta}+t \omega\frac{1}{2} + t (u_{\theta} - \omega) \\ &\quad= - \frac{1}{2} t \omega \\ &\quad\neq0 = \bar{v}(t) - u_{\theta} , \quad t \in(0,T]. \end{aligned}$$

This argument assumes that $S_{\infty}[0] = 1/2$. If one instead defines $S_{\infty}[0] = 1$, then *v̄* would solve the limit problem.

Due to the properties of the firing rate function (41) the source term $q_{\beta}$ in (44) becomes discontinuous. This can be avoided by instead using the smooth version (8)–(9) but then the analysis of this example becomes much more involved.

## Discussion and Conclusions

If a Heaviside firing rate function is used, the model (1) may not only have several solutions, but the *initial-condition*-to-*solution* map for this problem can become discontinuous [9]. It is thus virtually impossible to develop reliable numerical methods which employ finite precision arithmetic for such problems. One can try to overcome this issue by

- Attempting to solve the ill-posed equation with symbolic computations.
- Regularize the problem.

To the best of our knowledge, present symbolic techniques are not able to handle strongly nonlinear equations of the kind (1), even when $\beta< \infty$. We therefore analyzed the approach b), using the straightforward regularization technique obtained by replacing the Heaviside firing rate function by a Lipschitz continuous mapping. This yields an equation which is within the scope of the Picard–Lindelöf theorem and standard stability estimates for ODEs. That is well-posed and, at least in principle, approximately solvable by numerical methods.

Our results show that the sequence $\{ \mathbf{u}_{\beta} \}$ of regularized solutions will have at least one convergent subsequence. The limit, **v**, of this subsequence will satisfy the integral/Volterra form (27) of the limit problem, provided that the set $Z(\mathbf{v})$, see (15), has zero Lebesgue measure. Unfortunately, it seems to be very difficult to impose restrictions which would guarantee that **v** obeys this threshold property, which we refer to as threshold simple. Also, the example presented in Sect. 7 shows that, if the limit **v** is not threshold simple, then this function may not solve the associated equation with a Heaviside firing rate function.

One could propose to overcome the difficulties arising when $\beta= \infty$ by always working with finite slope firing rate functions. This would potentially yield a rather robust approach, provided that the entire sequence $\{ \mathbf{u}_{\beta} \}$ converges, because increasing a large *β* would still guarantee that $\mathbf{u}_{\beta}$ is close to the unique limit **v**. However, the fact that different convergent subsequences of $\{ \mathbf{u}_{\beta} \}$ can converge to different solutions of the limit problem, as discussed in Sect. 6, suggests that this approach must be applied with great care. In addition, the error amplification in the steep firing rate regime can become extreme [9] and the accurate numerical solution of such models is thus challenging.

What are the practical consequences of our findings? As long as there does not exist very reliable biological information about the size of the steepness parameter *β* and the shape of the firing rate function $S_{\beta}$, it seems that we have to be content with simulating with various $\beta< \infty$. If one observes that $\mathbf{u}_{\beta}$ approaches a threshold advanced limit, as *β* increases, or that the entire sequence does not converge, the alarm bell should ring. All simulations with large *β* must use error control methods which guarantee the accuracy of the numerical solution—we must keep in mind that we are trying to solve an almost ill-posed problem.

In neural field equations one employs a continuous variable, e.g., $x \in \mathbb {R}$, instead of a discrete index $i \in\{1,2, \ldots, N \}$. The sum in (1) is replaced by an integral; see [1, 2, 6]. For each time instance $t \in[0,T]$ one therefore does not get a vector $\mathbf{u}_{\beta}(t) \in \mathbb {R}^{N}$, as for the point neural models analyzed in this paper, but a function $\mathbf {u}_{\beta}(x,t)$, $x \in \mathbb {R}$. That is, in neural field equations the object associated with each fixed $t \in[0,T]$ belongs to an infinite dimensional space. It is often a subtle task to generalize concepts and proofs from a finite to an infinite dimensional setting: It is thus an open problem whether the techniques and results presented in this paper can be adapted to neural field models.
